# Additional prognostic value of stress cardiovascular magnetic resonance for cardiovascular risk stratification after a cryptogenic ischemic stroke

**DOI:** 10.3389/fcvm.2022.956950

**Published:** 2022-09-14

**Authors:** Solenn Toupin, Théo Pezel, Francesca Sanguineti, Marine Kinnel, Thomas Hovasse, Thierry Unterseeh, Stéphane Champagne, Philippe Garot, Jérôme Garot

**Affiliations:** ^1^Siemens Healthcare France, Saint-Denis, France; ^2^Cardiovascular Magnetic Resonance Laboratory, Institut Cardiovasculaire Paris Sud, Hôpital Privé Jacques Cartier, Ramsay Santé, Massy, France; ^3^Department of Cardiology, Lariboisiere Hospital–APHP, Inserm UMRS 942, University of Paris, Paris, France

**Keywords:** cardiovascular magnetic resonance (CMR) imaging, cardiovascular events (CVE), stroke, stress testing cardiac imaging, ischemia

## Abstract

**Background:**

One-third of ischemic strokes are “cryptogenic” without clearly identified etiology. Although coronary artery disease (CAD) is the main cause of death after stroke, the interest in CAD screening in patients with cryptogenic stroke is still debated.

**Aim:**

The aim of the study was to assess the incremental prognostic value of stress cardiovascular magnetic resonance (CMR) beyond traditional risk factors for predicting cardiovascular events in patients with a prior cryptogenic ischemic stroke.

**Materials and methods:**

Between 2008 and 2021, consecutive patients with prior cryptogenic strokes referred for stress CMR were included and followed for the occurrence of major adverse cardiovascular events (MACEs), defined by cardiovascular death or non-fatal myocardial infarction (MI). Univariable and multivariable Cox regressions were performed to determine the prognostic value of unrecognized MI and silent ischemia.

**Results:**

Of 542 patients (55.2% male, mean age 71.4 ± 8.8 years) who completed the follow-up (median 5.9 years), 66 (12.2%) experienced MACE. Silent ischemia and unrecognized MI were detected in 18 and 17% of patients, respectively. Using Kaplan–Meier analysis, silent ischemia and unrecognized MI were associated with the occurrence of MACE [hazard ratio, HR: 8.43 (95% CI: 5.11–13.9); HR: 7.87 (95% CI: 4.80–12.9), respectively, *p* < 0.001]. In multivariable analysis, silent ischemia and unrecognized MI were independent predictors of MACE [HR: 8.08 (95% CI: 4.21–15.5); HR: 6.65 (95% CI: 3.49–12.7), respectively, *p* < 0.001]. After adjustment, stress CMR findings showed the best improvement in model discrimination and reclassification above traditional risk factors (C-statistic improvement: 0.13; NRI = 0.428; IDI = 0.048).

**Conclusion:**

In patients with prior cryptogenic stroke, stress CMR findings have an incremental prognostic value to predict MACE over traditional risk factors.

## Introduction

One-third of ischemic strokes are “cryptogenic” and remain without clearly identified etiology despite extensive diagnostic work-up. In the absence of a clear etiology, managing secondary stroke prevention is challenging, and patients with prior cryptogenic stroke have a 10-year death risk of 46% (1). Ischemic stroke and coronary artery disease (CAD) share common risk factors (2) and previous studies reported a prevalence of CAD of 20–41% in patients with prior stroke (3–6). Consistently, cardiovascular (CV) events constitute the main cause of death after stroke, with 5–39% of CV death during a 10-year follow-up (7). In addition, patients with a prior cryptogenic stroke have a 10-year 10% risk of experiencing acute coronary syndrome (1). Therefore, it could be relevant to detect occult CAD in patients with recent prior cryptogenic stroke, who might benefit from additional preventive interventions. Indeed, the CONFIRM study showed the relevant prognostic role of coronary computed tomography angiography (CCTA) in the detection of non-significant or significant atheroma, (8) which could lead to the introduction of statins in the current guidelines. However, current American and European guidelines do not recommend systematic screening for CAD by functional stress testing or CCTA in these patients with prior cryptogenic stroke, except in those with symptomatic angina, dyspnea, or high CV risk (9–12).

Stress cardiovascular magnetic resonance (CMR) imaging has emerged as a cost-effective modality for the diagnosis of CAD, (13) and for the risk stratification of CV events through the detection of both inducible ischemia and myocardial scar without ionizing radiation (14–16). Several studies have shown the long-term prognostic value of both silent ischemia and unrecognized myocardial infarction (MI) in patients with suspected or known CAD (16–18), and in patients without known CAD (19). Although recent studies suggest that stress CMR can predict CV events in asymptomatic patients at high cardiovascular risk (20, 21), there are no targeted prognostic data in patients with recent cryptogenic stroke.

The main objective of the study was to determine whether the detection of silent ischemia or unrecognized MI through vasodilator stress CMR can provide incremental prognostic value above traditional CV risk factors to predict CV events in a cohort of patients with recent prior cryptogenic stroke and without known CAD.

## Materials and methods

### Study population

Between December 2008 and January 2021, we conducted a single-center longitudinal study with a retrospective enrolment of consecutive asymptomatic patients with prior cryptogenic ischemic stroke without known CV disease, referred for vasodilator stress perfusion CMR. Because patients with prior ischemic stroke carry a high risk of future coronary events, all patients presenting with prior cryptogenic ischemic stroke without known CV disease and ≥ 2 cardiovascular risk factors were referred to stress CMR for the screening of obstructive CAD. Recent cryptogenic ischemic stroke, occurring < 3 months before the CMR examination, was defined by an imaging-confirmed stroke (by cerebral magnetic resonance or computed tomography imaging) with an unknown source despite thorough diagnostic assessment (including at least carotid and cerebral arterial imaging, echocardiography and extended rhythm monitoring) (22). Patients were included if they had ≥ 2 coronary risk factors including age > 50 years for men or > 60 years for women, diabetes mellitus, hypertension, smoking, dyslipidemia, family history of CAD, and obesity defined by body mass index ≥ 30 kg/m^2^. Patients with a known stenosis ≥ 50% on at least one epicardial coronary artery on invasive coronary angiography or computed tomography angiography, patients with a positive functional test, patients with a history of revascularization (defined by previous percutaneous coronary intervention or coronary artery bypass graft), patients with prior MI, history of atrial fibrillation (AF), history of peripheral atheroma, prior hospitalization for heart failure or known left ventricular (LV) dysfunction [defined by LV ejection fraction (LVEF) < 50%] were excluded. Patients with any reported CV-related symptoms, such as chest pain or shortness of breath at rest or on exertion 6 months to enrollment, were also excluded. The absence of symptoms was confirmed by a senior cardiologist on the day of stress CMR. Other exclusion criteria are shown in Supplementary file 1. Clinical data were collected according to medical history and clinical examination on the day of stress CMR. Following the standard of care, all patients with inducible ischemia during the stress CMR exam were referred for invasive coronary angiography, whereas all patients with unrecognized MI but without inducible ischemia received optimal medical treatment. All patients provided informed written consent. The study was approved by the local Ethic Committee of our institutions (Hôpital privé Jacques Cartier, Ramsay Santé, Massy, France) and conducted in accordance with the 1964 Declaration of Helsinki. This study followed the Strengthening the Reporting of Observational Studies in Epidemiology (STROBE) reporting guideline for cohort studies.

### Patient follow-up and clinical outcomes

The follow-up consisted of a clinical visit as part of usual care (65%) or by direct contact with the subject or the referring cardiologist (35%). A clinical questionnaire with a detailed description of clinical study outcomes was filled out by three senior cardiologists. Data collection ended in January 2021. The primary outcome was the occurrence of at least one of the combined major adverse clinical events (MACEs) defined as CV mortality or non-fatal MI. The secondary outcome was CV mortality. Clinical event adjudication was based on the follow-up clinical visit or contact, with a consensus reached by two senior cardiologists. Non-fatal MI was defined by typical angina of ≥ 20 min duration, electrocardiogram changes, and a rise in troponin or creatine kinase level above the 99 percentile of the upper reference limit (23). CV mortality was defined as sudden cardiac death with documented fatal arrhythmias, or any death immediately preceded by acute MI, acute, or exacerbation of heart failure (HF), or stroke. All clinical events were defined according to the published standardized definitions (24). In patients with multiple events, only the first event was considered for event-free survival analysis. According to guidelines, hospitalization for HF was defined by symptoms and/or signs of HF with the evidence of diastolic or systolic dysfunction by echocardiography and elevated levels of natriuretic peptide (BNP > 35 pg/ml and/or NT-proBNP > 125 pg/ml) (25). In six patients who underwent percutaneous coronary intervention < 90 days after the index CMR examination, peri-procedural events (MI or CV mortality) (26) were not included in the analysis.

### Cardiovascular magnetic resonance protocol

All patients underwent CMR in a dedicated CMR laboratory using 1.5T scanners (MAGNETOM Espree, and MAGNETOM Aera, Siemens Healthcare, Erlangen, Germany). Detailed CMR protocol has been previously described (19, 20, 27). Briefly, long-axis and short-axis views covering the entire LV were obtained using a balanced steady-state free-precession sequence (b-SSFP). Vasodilatation was induced with dipyridamole injected at 0.84 mg/kg over 3 min for all patients. At the end of dipyridamole infusion, a bolus of gadolinium-based contrast agent (Dotarem, Guerbet, France, 0.1 mmol/kg) was injected at a rate of 5.0 ml/s. Stress perfusion imaging was performed using a saturation-prepared b-SSFP sequence with the following typical parameters: repetition time/echo time = 287/1.2 ms, acceleration factor = 2, field of view = 370 × 314 mm, and reconstructed voxel size = 1.7 × 1.7 × 8 mm. A series of six slices (four short-axis views, in addition to 2-and 4-chamber views) were acquired every other heartbeat. Then, theophylline was injected intravenously (250 mg over 5 min) to null the effect of dipyridamole. Ten minutes after contrast injection, single-breath-hold 3D T1-weighted inversion recovery gradient-echo sequence was acquired with the same prescriptions to detect late gadolinium enhancement (LGE). The inversion time was individually adjusted to null normal myocardium. In case of artifacts on LGE images, additional 2D single-shot b-SSFP images with phase-sensitive inversion recovery reconstruction were acquired. Patients were asked to refrain from caffeine at least 12 h before CMR. Safety was studied with clinical monitoring 1 h after CMR to assess major adverse events. A 12-lead electrocardiogram was performed both before and after the CMR examination.

### Cardiovascular magnetic resonance analysis

Left ventricular (LV) volumes and LVEF were calculated from the short-axis cine images (syngo.via, Siemens Healthcare, Erlangen, Germany). Stress perfusion and LGE images were evaluated according to the 17-segment model of the American Heart Association (28). The analysis of perfusion images was done visually by two experienced cardiologists (JG and FS) blinded to clinical and follow-up data. Silent ischemia was defined as a subendocardial perfusion defect that (1) occurred in at least one myocardial segment, (2) persisted for at least three phases beyond peak contrast enhancement, (3) followed a coronary distribution, and (4) occurred in the absence of co-localized LGE in the same segment (29–32). An unrecognized MI was defined by LGE with ischemic patterns defined by subendocardial or transmural LGE (33). The total number of ischemic segments was calculated for each patient. LGE was semi-quantitatively assessed using the number of LGE segments. All clinical and CMR characteristics were reported in a dedicated database (Hemolia, Clinigrid Inc., Paris, France).

### Statistical analysis

Continuous variables were expressed as mean ± standard deviation (SD) and categorical variables as the frequency with percentage. Follow-up was presented as the median and interquartile range (IQR). Differences between patients with and without silent ischemia in terms of baseline clinical and CMR characteristics were compared using the Student’s *t*-test or the Wilcoxon rank-sum test for continuous variables and the chi-square or Fisher’s exact test for categorical variables, as appropriate. Normal distribution was assessed using the Shapiro–Wilk test. Cumulative incidence rates of individual and composite outcomes were estimated using the Kaplan–Meier method and compared with the log-rank test. The proportional hazard assumption was visually assessed using Schoenfeld residuals. Data on patients who were lost to follow-up were censored at the time of the last contact. Cox proportional hazards methods were used to identify the predictors of MACE among patients with or without silent ischemia, and with or without unrecognized MI. The assumption of proportional hazards ratio (HR) was verified.

The different multivariable models used for adjustment were as follows:

Model 1: used a stepwise forward Cox regression strategy to select the strongest parsimonious set of clinical covariates for MACE and cardiovascular mortality, considering all clinical covariates with a *p*-value ≤ 0.2 on univariable screening (without the presence of silent ischemia and unrecognized MI).

Model 2: model 1 + presence of unrecognized MI.

Model 3: model 2 + presence of silent ischemia.

The discriminative capacity of each model for predicting MACE was determined according to Harrell’s C-statistic at baseline and after the addition of silent ischemia and unrecognized MI. The additional predictive value of the presence of silent ischemia and unrecognized MI was calculated by Harrell’s C-statistic increment, the continuous net reclassification improvement (NRI), and the integrative discrimination index (IDI) (34). NRI and IDI were computed at the end of follow-up using the R package “survIDINRI” (35).

To assess the clinical interest of stress CMR in patients with prior cryptogenic ischemic stroke, the prognostic value of stress CMR in this cohort was compared to a control population without CV disease from our center using a 1:1 propensity score-matched population (with vs. without prior cryptogenic ischemic stroke). A multivariable logistic regression model was built to estimate a propensity score for prior cryptogenic ischemic stroke, using the following variables: age, gender, and traditional cardiovascular risk factors. Imbalances between groups were considered to be small when the absolute standardized difference for a given covariate was less than 10%. The probit model with 1-to-1 nearest neighbor matching and without replacement was used to identify one patient without prior cryptogenic ischemic stroke (*N* = 542) for each patient with prior cryptogenic ischemic stroke (*N* = 542) (Supplementary file 2). The association between the presence of ischemia and the occurrence of MACE in the matched groups was assessed using a Cox proportional hazards regression model.

The prognostic value of silent ischemia in different subsamples of clinical interest was investigated by a forest plot. A two-tailed *p*-value < 0.05 was considered statistically significant. Statistical analysis was performed using R software, version 3.3.1 (R Project for Statistical Computing).

## Results

### Patient characteristics

**FIGURE 1:**
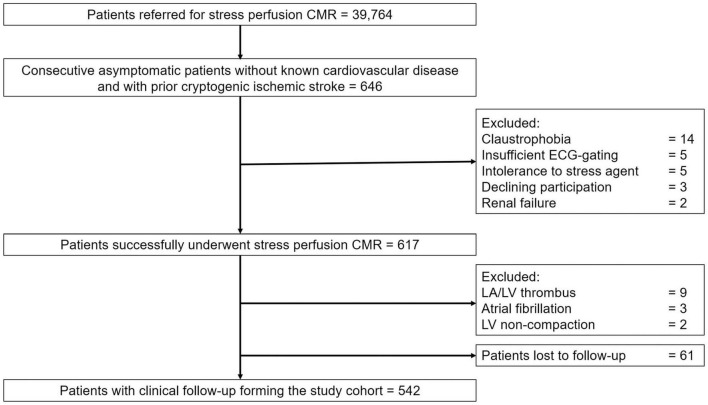
Study flowchart. CMR, cardiovascular magnetic resonance; ECG, electrocardiogram; LA, left atrium; LV, left ventricle.

From the initial cohort of 646 consecutive patients with prior cryptogenic stroke referred to stress CMR, 617 (83.9%) patients successfully completed the stress CMR examination. Reasons for failure to complete CMR are detailed in the study flowchart (Figure 1). No patient died during or shortly after CMR and there was one case of unstable angina. Detailed safety results are shown in Supplementary file 3. Fourteen patients (2.3%) were excluded because of CMR findings that were concordant with stroke etiology: left atrial or LV thrombus in nine patients (64%), atrial fibrillation in three patients (21%), and LV non-compaction in two patients (15%). Overall, 542 patients (83.9%) completed the clinical follow-up and constituted the study cohort.

Baseline patient characteristics and CMR findings stratified by the presence of silent ischemia are shown in Table 1. Twenty-two percent of patients had two CV risk factors, 38% had three CV risk factors, and 39% had ≥ 4 CV risk factors. The mean LVEF was 62.7 ± 10.0%. Silent ischemia was detected in 97 patients (18%) and unrecognized MI in 91 (17%). Thirty-four patients (4.8%) had both silent ischemia and unrecognized MI, and 27% of patients with unrecognized MI had silent ischemia. A total of 162 (30%) patients had an abnormal CMR exam, defined by the presence of silent ischemia and/or unrecognized MI. Patients with silent ischemia had a greater body mass index (26.0 ± 2.4 vs. 27.0 ± 3.3, *p* < 0.001), a higher rate of diabetes (62.9% vs. 44.7%, *p* = 0.002), and had a higher 10-year risk of fatal CAD (3.0% vs. 2.3%, *p* < 0.001) than patients without silent ischemia. Other coronary risk factors including hypertension, dyslipidemia, smoking, and family history of CAD, were similar between the two groups.

**TABLE 1:** Baseline and cardiovascular magnetic resonance (CMR) characteristics of patients with cryptogenic stroke with or without silent ischemia (*N* = 542).

	All patients (*N* = 542)	Without ischemia (*N* = 445)	With ischemia (*N* = 97)	*P*-value
Demographics				
Age, years	71.4 ± 8.8	71.4 ± 8.8	71.4 ± 8.5	0.982
Male, n (%)	299 (55.2)	242 (54.4)	57 (58.8)	0.501
Body mass index, kg/m^2^	26.8 ± 3.2	27.0 ± 3.3	26.0 ± 2.4	**< 0.001**
Coronary risk factors, n (%)				
Diabetes mellitus	260 (48.0)	199 (44.7)	61 (62.9)	**0.002**
Hypertension	402 (74.2)	333 (74.8)	69 (71.1)	0.531
Dyslipidemia	316 (58.3)	254 (57.1)	62 (63.9)	0.261
Current or previous smoking	177 (32.7)	143 (32.1)	34 (35.1)	0.663
Family history of CAD	45 (8.3)	42 (9.4)	3 (3.1)	0.064
Ten-year risk for fatal CAD (%)*	2.4 (0.8–5.6)	2.3 (0.7–5.5)	3.0 (1.0–6.0)	**< 0.001**
Stress CMR				
LV ejection fraction,%	62.7 ± 10.0	62.8 ± 9.0	62.0 ± 13.6	0.546
LV end-diastolic volume index, ml/m^2^	62.7 ± 13.6	62.3 ± 12.6	64.8 ± 17.7	0.185
LV end-systolic volume index, ml/m^2^	23.0 ± 5.2	23.1 ± 5.2	22.6 ± 5.4	0.466
Presence of unrecognized MI, n (%)	91 (16.8)	65 (14.6)	26 (26.8)	**0.006**
Number of segments of LGE	0.4 ± 1.1	0.3 ± 0.8	0.8 ± 1.6	**< 0.001**
Number of segments of ischemia	0.4 ± 1.0	0.0 ± 0.0	2.1 ± 1.6	**< 0.001**
RPP at baseline, mmHg/beats/min	9.1 (7.0–11.3)	9.1 (7.0–11.3)	9.2 (7.1–11.4)	0.861
RPP at stress, mmHg/beats/min	10.5 (8.1–12.6)	10.4 (8.1–12.4)	11.1 (9.3–13.5)	0.671

Values are n (%), mean ± SD, or median (interquartile range). *Based on a modified SCORE project (https://www.escardio.org/Education/Practice-Tools/CVD-prevention-toolbox/SCORE-Risk-Charts) that did not take into account the total cholesterol level. BMI, body mass index; CAD, coronary artery disease; CMR, cardiac magnetic resonance; LGE, late gadolinium enhancement; LV, left ventricle; MI, Myocardial infarction; RPP, rate-pressure product (pressure mmHg x Heart rate bpm)/1000; SD, standard deviation. Bold values mean that the P values reached statistical significance (P < 0.005).

Using the propensity score-matched populations (with vs. without prior cryptogenic ischemic stroke), patients with prior cryptogenic ischemic stroke had a higher rate of both silent ischemia (17.9 vs. 14%) and unrecognized MI (16.8 vs. 12.2%, both *p* < 0.001) than patients without prior cryptogenic ischemic stroke (Supplementary file 2).

### Primary outcome

**FIGURE 2:**
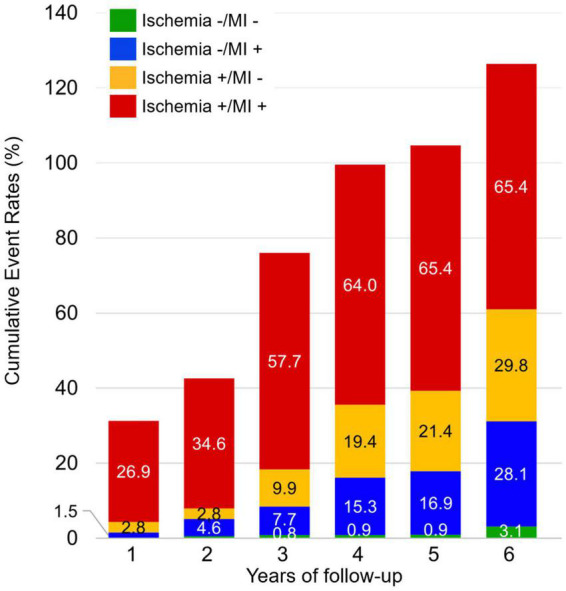
Cumulative rates of major adverse cardiovascular events (MACE) during follow-up stratified by the presence of silent ischemia and unrecognized myocardial infarction (MI).

The median (IQR) follow-up duration was 5.9 years (4.3–6.3 years). Of the 542 patients, 66 (12.2%) experienced a MACE, including 39 (7.2%) CV deaths and 27 non-fatal MI (5.0%). The annualized rate of MACE was 3.7%/year. The cumulative rate of MACE was higher in patients with both silent ischemia and unrecognized MI than in patients without ischemia or MI (*p* < 0.001). Patients with unrecognized MI but without silent ischemia had a similar cumulative rate of MACE than patients with silent ischemia but without unrecognized MI (non-significant *p*-value) (Figure 2).

**FIGURE 3:**
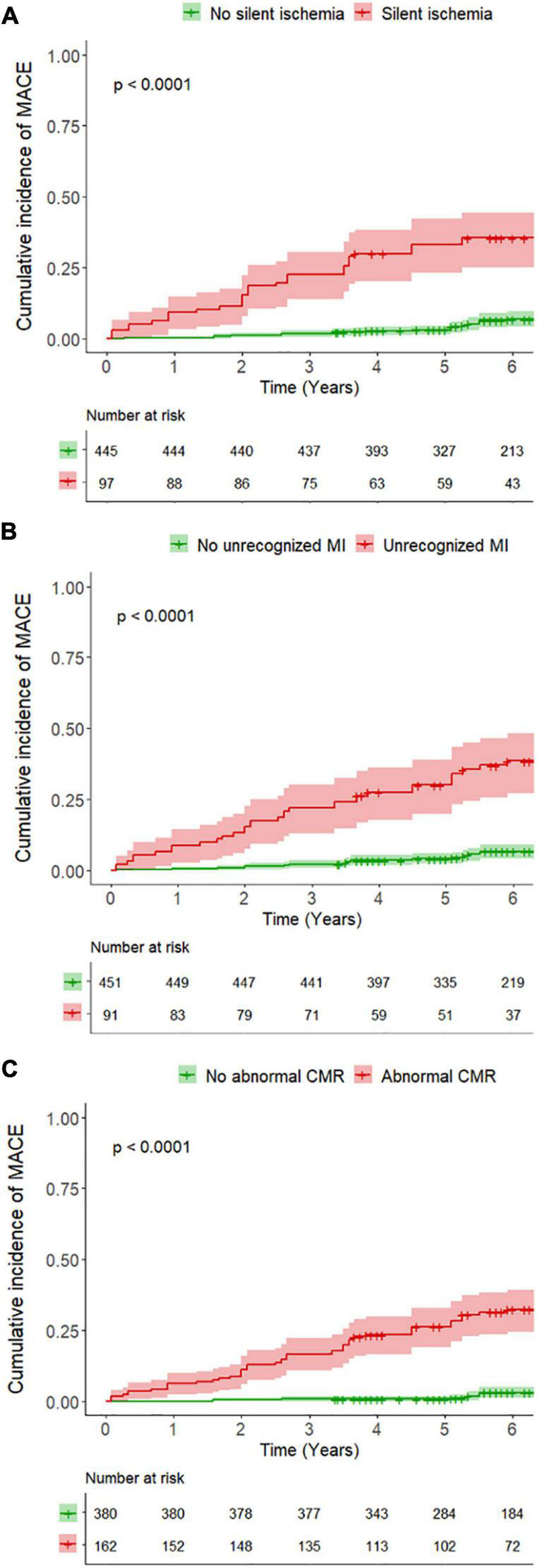
Kaplan–Meier curves for major adverse cardiovascular events (MACE) stratified by **(A)** the presence of silent ischemia, **(B)** the presence of unrecognized myocardial infarction (MI), and **(C)** the presence of an abnormal cardiovascular magnetic resonance (CMR) (silent ischemia or unrecognized MI). Test comparing the two groups is based on the log-rank test.

**FIGURE 4:**
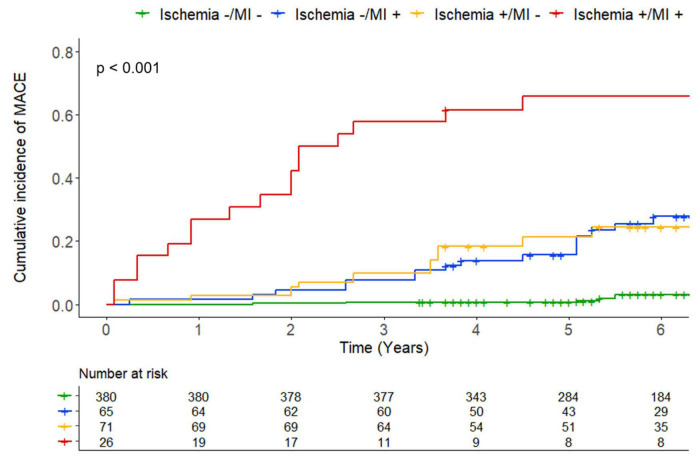
Kaplan–Meier curves for major adverse cardiovascular events (MACE) in four subgroups: patients without ischemia and myocardial infarction (MI), with MI, and without ischemia, with ischemia and without MI, and with both ischemia and MI. Test comparing the two groups is based on the log-rank test.

In univariable analysis, age, diabetes mellitus, hypertension, LVEF, presence and extent of silent ischemia, presence and extent of unrecognized MI, and abnormal CMR were all significantly associated with MACE (Table 2 and Supplementary file 4). Using Kaplan–Meier analysis, silent ischemia, unrecognized MI, and abnormal CMR (ischemia or MI) were all associated with the occurrence of MACE (HR: 8.43; 95% CI: 5.11 to 13.9; HR: 7.87; 95% CI: 4.80 to 12.9; and HR: 15.9; 95% CI: 7.88 to 32.3; all *p* < 0.001, respectively) (Figure 3). The cumulative incidence of MACE stratified by the presence of silent ischemia and/or unrecognized MI is shown in Figure 4. In addition, silent ischemia was also associated with CV mortality (HR: 8.08; 95% CI: 4.21 to 15.5; *p* < 0.001), non-fatal MI (HR: 8.99; 95% CI: 4.11 to 19.7; *p* < 0.001), late coronary revascularization (HR: 3.31; 95% CI: 1.30 to 8.41; *p* = 0.012), and all-cause of mortality (HR: 2.33; 95% CI: 1.45 to 3.72; *p* < 0.001) (Supplementary file 5). The prognostic value of silent ischemia remained consistent in all other subsamples of clinical interest such as men and women, diabetics and non-diabetics, regardless of LVEF (Supplementary file 6).

**TABLE 2:** Univariable analysis of clinical and cardiac magnetic resonance (CMR) characteristics for the prediction of adverse events.

	MACE		Cardiovascular mortality	
		
	Hazard ratio (95% CI)	*P*-value	Hazard ratio (95% CI)	*P*-value
Age	1.02 (1.01–1.03)	**0.041**	1.05 (1.01–1.10)	**0.022**
Male	1.05 (0.64–1.71)	0.852	0.98 (0.52–1.86)	0.947
Body mass index	1.04 (0.97–1.11)	0.334	1.00 (0.91–1.10)	0.985
Diabetes mellitus	2.91 (1.69–5.01)	**< 0.001**	3.61 (1.71–7.63)	**0.001**
Hypertension	1.68 (1.27–2.21)	**< 0.001**	1.51 (1.23–2.04)	**< 0.001**
Dyslipidemia	1.00 (0.61–1.64)	0.989	1.40 (1.19–1.79)	0.022
Current or previous smoking	1.38 (0.84–2.27)	0.202	0.54 (0.25–1.18)	0.121
Family history of CAD	1.43 (0.65–3.13)	0.373	1.84 (0.72–4.73)	0.203
LVEF, per 10%	0.78 (0.64–0.97)	**0.023**	0.92 (0.67–1.26)	0.603
LV end-diastolic volume index, per 10 ml/m^2^	0.98 (0.81–1.14)	0.819	0.82 (0.64–1.06)	0.134
LV end-systolic volume index, per 10 ml/m^2^	1.05 (0.65–1.70)	0.838	0.64 (0.36–1.15)	0.136
Presence of unrecognized MI	7.87 (4.80–12.9)	**< 0.001**	6.65 (3.49–12.7)	**< 0.001**
Number of segments with unrecognized MI	2.19 (1.95–2.47)	**< 0.001**	2.28 (1.95–2.66)	**< 0.001**
Presence of silent ischemia	8.43 (5.11–13.9)	**< 0.001**	8.08 (4.21–15.5)	**< 0.001**
Number of segments with ischemia	1.65 (1.49–1.83)	**< 0.001**	1.57 (1.36–1.82)	**< 0.001**
Abnormal CMR	15.9 (7.88–32.3)	**< 0.001**	11.2 (4.94–25.6)	**< 0.001**

Abbreviations: Same as in Table 1. CI, confidence interval; LVEF, left ventricular ejection fraction; MACE, major adverse cardiac events. Bold values mean that the P values reached statistical significance (P < 0.005).

**FIGURE 5:**
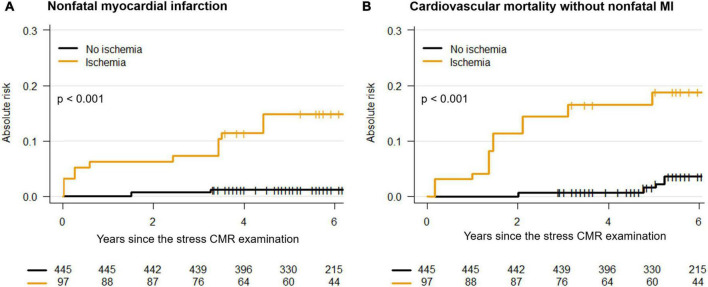
Competing risk analysis. Cumulative incidence functions of non-fatal myocardial infarction (MI) **(A)** and cardiovascular mortality without non-fatal MI **(B)**.

In multivariable stepwise Cox regression (model 3), the presence of ischemia and unrecognized MI were independent predictors of a higher incidence of MACE (HR = 12.4; 95% CI: 6.62 to 23.2, and HR = 9.53; 95% CI: 5.49 to 16.5, both *p* < 0.001, respectively) and CV mortality (HR = 12.1; 95% CI: 5.11 to 28.7, and HR = 12.0; 95% CI: 5.60 to 25.9, both *p* < 0.001, respectively) (Table 3). In competing for risk analysis, the presence of silent ischemia was independently associated with non-fatal MI and CV mortality (both *p* < 0.001) (Figure 5 and Table 4).

**TABLE 3:** Multivariable cox regression analysis for the prediction of adverse events.

	MACE		Cardiovascular mortality	
		
	Hazard ratio (95% CI)	*P*-value	Hazard ratio (95% CI)	*P*-value
Model 1*				
Age	1.00 (0.97–1.03)	0.813	1.06 (1.03–1.11)	**0.033**
Male	0.94 (0.57–1.54)	0.797	0.79 (0.41–1.56)	0.500
Body mass index	1.05 (0.97–1.13)	0.229	1.06 (0.95–1.17)	0.195
Diabetes mellitus	2.79 (1.51–5.15)	**< 0.001**	2.61 (1.42–5.55)	**< 0.001**
Hypertension	1.51 (1.23–2.04)	**< 0.001**	3.06 (1.34–7.00)	**0.008**
Dyslipidemia	0.61 (0.35–1.07)	0.083	1.36 (1.18–1.72)	**0.004**
Current or previous smoking	1.29 (0.76–2.17)	0.346	0.53 (0.24–1.18)	0.121
LVEF, per 10%	0.74 (0.59–0.93)	**0.011**	1.05 (0.72–1.54)	0.781
Model 2^†^				
Presence of unrecognized MI	8.44 (4.94–14.4)	**< 0.001**	9.40 (4.59–19.2)	**< 0.001**
Model 3^‡^				
Presence of unrecognized MI	9.53 (5.49–16.5)	**< 0.001**	12.0 (5.60–25.9)	**< 0.001**
Presence of silent ischemia	12.4 (6.62–23.2)	**< 0.001**	12.1 (5.11–28.7)	**< 0.001**

*Covariates in model 1 by stepwise variable selection with entry and exit criteria set at the p ≤ 0.2 level: -For MACE: age, male, hypertension, family history of CAD, LVEF per 10%, LV end-systolic volume index, per 10 ml/m^2^. -For CV mortality: age, male, family history of CAD, family history of CAD. †Covariates in model 2: model 1 with unrecognized MI. ‡Covariates in model 3: model 2 with silent ischemia. BMI, body mass index; CAD, coronary artery disease; CI, confidence interval; CV, cardiovascular; LGE, late gadolinium enhancement; MACE, major adverse cardiac events; LV, left ventricle; LVEF, left ventricular ejection fraction; MI, myocardial infarction. Bold values mean that the P values reached statistical significance (P < 0.005).

**TABLE 4:** Univariable and multivariable competing risk regression analyses (*N* = 542).

	Non-fatal MI				Cardiovascular mortality			
		
	Univariable analysis		Multivariable analysis^†^		Univariable analysis		Multivariable analysis^†^	
	
	sHR* (95% CI)	*P*-value	sHR* (95% CI)	*P*-value	sHR* (95% CI)	*P*-value	sHR* (95% CI)	*P*-value
Age	1.02 (1.01–1.06)	**0.002**	1.07 (0.99–1.15)	0.061	1.07 (1.01–1.13)	**0.015**	1.06 (0.99–1.13)	0.079
Male	1.21 (0.50–2.96)	0.670	-	-	0.83 (0.41–1.67)	0.610	-	-
Body mass index	1.12 (1.06–1.18)	**< 0.001**	1.01 (0.86–1.18)	0.910	1.03 (0.93–1.14)	0.580	-	-
Hypertension	1.22 (1.16–2.18)	**0.021**	1.20 (0.77–2.03)	0.710	1.53 (0.64–3.68)	0.340	-	-
Diabetes	4.39 (1.46–13.2)	**0.008**	4.30 (1.43–12.9)	**0.009**	4.55 (1.87–11.0)	**0.001**	-	-
Dyslipidemia	1.27 (0.49–3.28)	0.620	-	-	0.65 (0.32–1.30)	0.220	-	-
Smoking	3.89 (1.57–9.63)	**0.003**	1.31 (0.88–1.91)	0.620	0.70 (0.31–1.58)	0.400	-	-
LVEF, per 10%	0.95 (0.92–0.98)	**0.001**	0.93 (0.88–0.98)	**0.005**	1.00 (0.96–1.03)	0.930	-	-
Presence of silent ischemia	14.1 (5.20–32.4)	**< 0.001**	8.89 (4.10–18.1)	**< 0.001**	7.50 (3.63–15.5)	**< 0.001**	8.30 (3.53–19.5)	**< 0.001**
Presence of unrecognized MI	5.04 (2.06–12.3)	**< 0.001**	4.01 (1.78–9.61)	**< 0.001**	4.17 (2.08–8.37)	**< 0.001**	3.97 (1.66–9.41)	**< 0.001**

*HR of the subdistribution hazard function. †Covariates by stepwise variable selection with entry and exit criteria set at the p < 0.20 level; for non-fatal MI: age, body mass index, hypertension, diabetes mellitus, smoking, LVEF, presence of unrecognized MI, and presence of silent ischemia; for cardiovascular mortality: age, diabetes mellitus, presence of unrecognized MI, and presence of silent ischemia. Abbreviations: Same as in Table 2. HR, hazard ratio. Bold values mean that the P values reached statistical significance (P < 0.005).

Using propensity score-matching, the prognostic value of silent ischemia (HR = 12.4; 95% CI: 6.62 to 23.2 vs. HR = 3.72; 95% CI: 2.75 to 5.22, *p* < 0.001) and unrecognized MI (HR = 9.53; 95% CI: 5.49 to 16.5 vs. HR = 1.77; 95% CI: 1.29 to 2.57, *p* < 0.001) to predict the occurrence of MACE was greater in patients with prior cryptogenic ischemic stroke compared to patients without prior cryptogenic ischemic stroke (Supplementary file 7).

### Incremental prognostic value of silent ischemia and unrecognized myocardial infarction

For the prediction of MACE, C-statistic values were 0.75 (95% CI: 0.70 to 0.79) for “model 1” with traditional risk factors built by stepwise variable selection. The addition of unrecognized MI significantly improved the C-statistic value to 0.84 (95% CI: 0.78 to 0.88; C-statistic improvement for “model 1”: 0.09; NRI = 0. 258; IDI = 0.036). The addition of both unrecognized MI and silent ischemia significantly improved the C-statistic value to 0.88 (95% CI: 0.84 to 0.91; C-statistic improvement for “model 1”: 0.13; NRI = 0.428; IDI = 0.048) (Table 5).

**TABLE 5:** Discrimination and reclassification associated with silent ischemia and unrecognized myocardial infarction (MI) for the prediction of major adverse cardiovascular events (MACE).

	MACE		
	
	C-index (95% CI)	NRI (95% CI)	IDI (95% CI)
Model 1 (stepwise selection)*	0.75 (0.70–0.79)	Reference	Reference
Model 2 (model 1 + unrecognized MI)^†^	0.84 (0.78–0.88)	0.258 (0.071–0.445)	0.036 (0.015–0.057)
Model 3 (model 2 + ischemia)^‡^	0.88 (0.84–0.91)	0.428 (0.209–0.647)	0.048 (0.023–0.068)

*Covariates in model 1 by stepwise variable selection with entry and exit criteria set at the p ≤ 0.2 level: age, male, hypertension, family history of CAD, LVEF per 10%, LV end-systolic volume index, per 10 ml/m^2^. †Covariates in model 2: model 1 with unrecognized MI. ‡Covariates in model 3: model 2 with silent ischemia. Abbreviations: Same as in Table 2. IDI, integrative discrimination index; NRI, net reclassification improvement.

For the prediction of CV mortality, C-statistic values were 0.74 (95% CI: 0.68 to 0.79) for “model 1” with traditional risk factors built by stepwise variable selection. The addition of unrecognized MI significantly improved the C-statistic value to 0.80 (95% CI: 0.72 to 0.86; C-statistic improvement for “model 1”: 0.06). The addition of both unrecognized MI and silent ischemia significantly improved the C-statistic value to 0.85 (95% CI: 0.80 to 0.89; C-statistic improvement for “model 1”: 0.11).

## Discussion

In a population of high CV risk patients with recent prior cryptogenic stroke, with high cardiovascular risk but without known CAD, referred for stress CMR, the main findings are: (1) 18% of patients had silent ischemia and 17% had unrecognized MI; (2) both silent ischemia and unrecognized MI were independent long-term predictors of MACE and CV mortality; and (3) the presence of silent ischemia and unrecognized MI improved model discrimination and reclassification for the prediction of MACE above traditional risk factors.

### Prevalence of occult coronary artery disease in patients with cryptogenic stroke

In asymptomatic patients without known CAD, the prevalence of occult CAD was substantial in the current study, with 30% of patients having silent ischemia or unrecognized MI. This finding is in line with the Asymptomatic Myocardial Ischemia in Stroke and Atherosclerotic Disease (AMISTAD) study, in which the prevalence of obstructive CAD (stenosis ≥ 50% by invasive coronary angiography) was 26% in ischemic stroke patients without known CAD (5). Recent studies using CCTA reported rates of significant CAD (at least 1 ≥ 50% stenosis) up to 18–48% in patients with stroke (4, 6, 36–38). In the current study, the prevalence of silent ischemia was lower (18%), but angiography and CCTA may be inaccurate in assessing the functional significance of coronary stenosis (14, 39), and a ≥ 50% stenosis does not imply functional significance. However, it is important to emphasize the prognostic role of CCTA for detecting non-significant coronary plaques reflecting a higher risk of CV events that may guide the prescription of statins (8). In addition, recent studies have shown the interest in CCTA for the detection of vulnerable coronary plaques at risk for acute coronary syndrome (40, 41). Consistently, the prevalence of silent ischemia is also in line with recent studies of stress CMR in asymptomatic patients, which reported a prevalence of 15% in patients without known CAD (20), and 28% in patients with prior known CAD (19). Similar to the Stress CMR Perfusion Imaging in the United States (SPINS) study, about one-third of patients with unrecognized MI also had silent ischemia (18).

The prevalence of unrecognized MI of 17% in the current study is also consistent with prior studies using CMR to detect LGE in stroke patients. Haeusler et al. reported a rate of unrecognized MI up to 15% among 89 patients with prior cryptogenic stroke, and 85% of them had no history of CAD (42). In agreement, a prevalence of 22% of unrecognized MI was recently reported in a series of patients with ischemic stroke assessed by cerebral and CMR (43).

Whether patients with ischemic stroke should be screened for asymptomatic CAD remains debated. In our study, the relatively high incidence rates of MACE and CV mortality (12.2 and 7.2% during a median follow-up of 5.9 years, respectively) support a strategy of accurate risk stratification for CAD in patients with prior cryptogenic stroke.

### Risk stratification of asymptomatic patients with prior cryptogenic stroke

In agreement with prior studies (19–21), the current data show that stress CMR has an accurate prognostic value for predicting MACE and CV mortality in patients with prior cryptogenic stroke, with the excellent safety profile. In line with others (18), unrecognized MI was also independently associated with the occurrence of MACE in those patients and improved the prediction risk model of MACE over traditional risk factors. Although some studies show a higher risk of MACE for patients with unrecognized MI without ischemia than patients with ischemia without unrecognized MI (15), our study described no significant difference between these two groups at 6 years of follow-up (MACE rate: 28.1 vs. 29.8%, respectively, *p* = 0.71). Using propensity score-matching, the prognostic value of the presence of silent ischemia and unrecognized MI was more than 3-fold higher in patients with prior cryptogenic ischemic stroke compared to patients without prior cryptogenic ischemic stroke after adjustment for traditional risk factors. All these findings suggest a relevant clinical interest in stress CMR for improved risk stratification in this specific population and may have implications for improved secondary prevention of patients with stroke. About one-third of ischemic strokes are cryptogenic, precluding targeted secondary prevention strategies (22). The American Heart Association/American Stroke Association recommends considering non-invasive testing for asymptomatic CAD in stroke patients with high cardiovascular risks (9–11). The current data showing a high burden of CAD (30%) in cryptogenic stroke patients without CV symptoms or history of CAD support the use of functional testing for the detection of CAD in those patients. Indeed, the incremental prognostic value of stress CMR could prove very useful to optimize secondary prevention strategies. Indeed, recent studies suggested the potential role of new therapies targeting coagulation and inflammation to decrease the risk of CV events in secondary prevention (44, 45). An improved risk stratification using stress CMR could allow to identify high-risk patients who could benefit from treatment intensification, new therapy, and/or revascularization. Although the quantification of myocardial blood flow (MBF) by CMR was not performed in the current study, several reports described its potential interest in accurately and reproducibly assessing the burden of ischemia to guide optimal therapy (46). Along with its added prognostic value, the steadily increasing expertise and availability of stress CMR make it a safe, reproducible, and reliable test to stratify the risk of cardiovascular events in asymptomatic patients with prior stroke and without known CAD.

### Study limitations

First, this study is a single-center study performed with a selected group of high CV risk patients with prior ischemic cryptogenic stroke referred to stress CMR. Patients without or with only one CV risk factor were not included, which may overestimate the prevalence of ischemia and select the patients at high risk for cardiovascular events. Thus, the current study does not assess the prognostic value of stress CMR in younger patients with ischemic cryptogenic stroke and fewer CV risk factors. This study does not reflect on whether stress CMR screening improved outcomes, and future randomized trials should be conducted to assess the impact of stress CMR screening on secondary prevention. Second, 61 (9.9%) patients were lost to follow-up, which can be explained by the relatively long follow-up and the design of the study. However, the French National Registry of Death has been carefully reviewed, which strengthens the mortality data. Third, only asymptomatic patients were included in the current study. In addition, arrhythmias were not collected during the follow-up. There are numerous clinical studies showing the diagnostic and prognostic accuracy of stress CMR in symptomatic patients with known or suspected CAD. Those studies included patients with prior strokes. For those reasons, symptomatic patients were excluded from the current study and the analysis focused on asymptomatic patients with recent cryptogenic stroke in whom risk stratification data were missing. Fourth, baseline and follow-up data for medications and ECG findings were not collected in the study. Consistently, the potential consequences on outcomes of the changes in decision-making (i.e., statin therapy, etc.) due to stress CMR findings could not be collected in this retrospective study. Fifth, the detection of myocardial ischemia on stress CMR images was only visual which may underestimate the amount of ischemia, but it represents the most widely used clinical method with optimal diagnostic accuracy. In addition, LGE quantification was performed using the number of segments without considering the transmural extent. Finally, dipyridamole was used as a stress agent mainly because of medico-economic reasons and a very close efficacy/safety profile compared to adenosine.

## Conclusion

Stress perfusion CMR has a good discriminative and incremental long-term prognostic value in asymptomatic high-risk patients with recent prior cryptogenic stroke but without known CAD. These data support the role of stress CMR for the screening of occult CAD in high-risk patients with recent cryptogenic stroke and at least two CV risk factors. Whether those findings could result in advances in decision-making and ultimately turn into clinical benefits needs further evaluation.

## Data availability statement

The raw data supporting the conclusions of this article will be made available by the authors, without undue reservation.

## Ethics statement

The studies involving human participants were reviewed and approved by local committee. The patients/participants provided their written informed consent to participate in this study.

## Author contributions

TP and JG conceived the study design. TP, FS, MK, TH, SC, TU, PG, and JG obtained the CMR images and analyzed the CMR scans. TP, ST, and JG analyzed the data and drafted the manuscript with critical revision. JG and ST had technically defined the CMR protocol. As authors, we attest to each of our substantial contributions to the manuscript and revision. All authors participated in the discussion of the concept of the study, read, and approved the final manuscript.

## Abbreviations

AF: atrial fibrillation
CAD: coronary artery disease
CCTA: coronary computed tomography angiography
CMR: cardiovascular magnetic resonance
CV: cardiovascular
IQR: interquartile range
HF: heart failure
HR: hazard ratio
LGE: late gadolinium enhancement
LV: left ventricular
LVEF: left ventricular ejection fraction
MACE: major adverse clinical events
MI: myocardial infarction.
