# Supplementary material for: Additional prognostic value of stress cardiovascular magnetic resonance for cardiovascular risk stratification after a cryptogenic ischemic stroke
Source: Front Cardiovasc Med. 2022 Sep 14;9:956950. doi: 10.3389/fcvm.2022.956950 (PMC9515378; doi:10.3389/fcvm.2022.956950)
Supplement: Supplementary file 1 [file Data_Sheet_1.docx]

**Supplemental Files**

**Table of Contents:**

- **Supplemental file 1.** List of exclusion criteria.
- **Supplemental file 2.** Baseline and CMR characteristics of the propensity-matched population and of patients with and without prior ischemic cryptogenic stroke.
- **Supplemental file 3.** Safety results.
- **Supplemental file 4.** Kaplan-Meier curves for MACE stratified by (A) the extent of silent ischemia, (B) the extent of unrecognized MI. Test comparing the two groups is based on the log-rank test.
- **Supplemental file 5**. Univariable and multivariable analyses of inducible myocardial ischemia for prediction of adverse events (N=542).
- **Supplemental file 6**. Subgroup analysis.
- **Supplemental file 7**. Multivariable Cox regression analysis for the prediction of MACE in patients with and without prior ischemic cryptogenic stroke using the propensity-matched population.

**SUPPLEMENTAL FILE 1**

**List of exclusion criteria**

Exclusion criteria were: (1) patients with a known stenosis ≥50% on at least 1 epicardial coronary artery on invasive coronary angiography or computed tomography angiography, patients with a positive functional test; (2) patients with a history of MI, defined by a history of MI on the medical records or presence of significant Q wave on 12-lead ECG in a coronary territory; (3) prior revascularization [percutaneous coronary intervention or coronary artery bypass graft]; (4) history of acute hospitalization for heart failure defined by the use of intravenous diuretics, or known LV dysfunction (defined by LVEF<50%); (5) known peripheral atheroma; (6) history of atrial fibrillation on the medical records or deﬁned by 12-lead electrocardiogram before and after CMR with consensus between 2 senior cardiologists; (7) any cardiovascular-related symptoms such as chest pain or shortness of breath at rest or on exertion 6 months prior to enrollment; (8) contraindication to CMR (cerebral clips, metallic eye implant); (9) contraindication to dipyridamole (severe asthma or chronic obstructive pulmonary disease, second- or third-degree atrioventricular block); (10) known cardiomyopathy (e.g. hypertrophic, dilated, or infiltrative) and acute or chronic myocarditis; (11) contraindications to CMR (cerebral clips, metallic eye implants); (12) contraindications to dipyridamole (severe asthma or chronic obstructive pulmonary disease, second- or third-degree atrioventricular block, recent decompensated heart failure); (13) known allergy to gadolinium-based contrast medium; and (14) glomerular filtration rate <30 mL/min/1.73 m^2^.

**SUPPLEMENTAL FILE 2**

**Baseline and CMR characteristics of the propensity-matched populations (patients with and without prior ischemic cryptogenic stroke)**

|  | **Patients**  **WITH**  **prior ischemic cryptogenic stroke**  **(N=542)** | **Propensity-matched patients**  **WITHOUT**  **prior ischemic cryptogenic stroke**  **(N=542)** | **p value** |
| --- | --- | --- | --- |
| Demographics |  |  |  |
| Age, years | 71.4 ± 8.8 | 71.3 ± 8.7 | 0.63 |
| Male, n (%) | 299 (55.2) | 299 (55.2) | 1.000 |
| Body mass index, kg/m² | 26.8 ± 3.2 | 27.3 ± 3.7 | **<0.001** |
|  |  |  |  |
| Coronary risk factors, n (%) |  |  |  |
| Diabetes mellitus | 260 (48.0) | 260 (48.0) | 1.000 |
| Hypertension | 402 (74.2) | 402 (74.2) | 1.000 |
| Dyslipidemia | 316 (58.3) | 315 (58.1) | 0.91 |
| Current or previous smoking | 177 (32.7) | 177 (32.7) | 1.000 |
| Family history of CAD | 45 (8.3) | 42 (7.7) | 0.78 |
|  |  |  |  |
| Ten-year risk for fatal CAD (%)* | 2.4 (0.8–5.6) | 2.4 (0.8–5.7) | 0.81 |

| Stress CMR |  |  |  |
| --- | --- | --- | --- |
| LV ejection fraction, % | 62.7 ± 10.0 | 68.9 ± 11.2 | **<0.001** |
| LV end-diastolic volume index, ml/m^2^ | 62.7 ± 13.6 | 60.1 ± 12.3 | **<0.001** |
| LV end-systolic volume index, ml/m^2^ | 23.0 ± 5.2 | 24.1 ± 5.0 | 0.08 |
| Presence of unrecognized MI, n (%) | 91 (16.8) | 66 (12.2) | **<0.001** |
| Number of segments of LGE | 0.4 ± 1.1 | 0.2 ± 1.0 | **<0.001** |
| Presence of ischemia | 97 (17.9) | 76 (14.0) | **<0.001** |
| Number of segments of ischemia | 0.4 ± 1.0 | 0.3 ± 0.9 | **<0.001** |
| RPP at baseline, mmHg/beats/min | 9.1 (7.0–11.3) | 8.9 (6.7–11.0) | **<0.001** |
| RPP at stress, mmHg/beats/min | 10.5 (8.1–12.6) | 10.2 (7.6–12.5) | **<0.001** |

*Values are n (%), mean ± SD, or median (interquartile range).*

*based on a modified SCORE project (https://www.escardio.org/Education/Practice-Tools/CVD-prevention-toolbox/SCORE-Risk-Charts) that did not take into account the total cholesterol level.

*Abbreviations: BMI: body mass index; CAD: coronary artery disease; CMR: cardiac magnetic resonance; LGE: late gadolinium enhancement; LV: left ventricle; MI: Myocardial infarction; RPP: rate-pressure product (pressure mmHg x Heart rate bpm)/1000; SD: standard deviation.*

**SUPPLEMENTAL FILE 3**

**Safety results**

There was one case of unstable angina, but no transient ischemic attack, disabling stroke, ST elevation MI or sustained ventricular tachycardia in relation to stress CMR.

Among the 617 patients completed the stress CMR protocol, the main adverse events during or immediately after the study were as follows: 88 headaches (14.3%), 65 chest discomforts due to dipyridamole (10.5%), 52 nausea or vomiting (8.4%), 11 angina with ECG evidence of ischemia (1.8%), and 8 dizziness (1.3%). For all patients, symptoms resolved quickly intravenous theophylline and with additional sublingual nitrates and/or intravenous betablockers in 13 patients (2.1%).

**SUPPLEMENTAL FILE 4**

**Figure. Kaplan-Meier curves for MACE stratified by (A) the extent of silent ischemia, and (B) the extent of unrecognized MI.**

Abbreviations: MACE: major adverse clinical events, MI: myocardial infarction

**
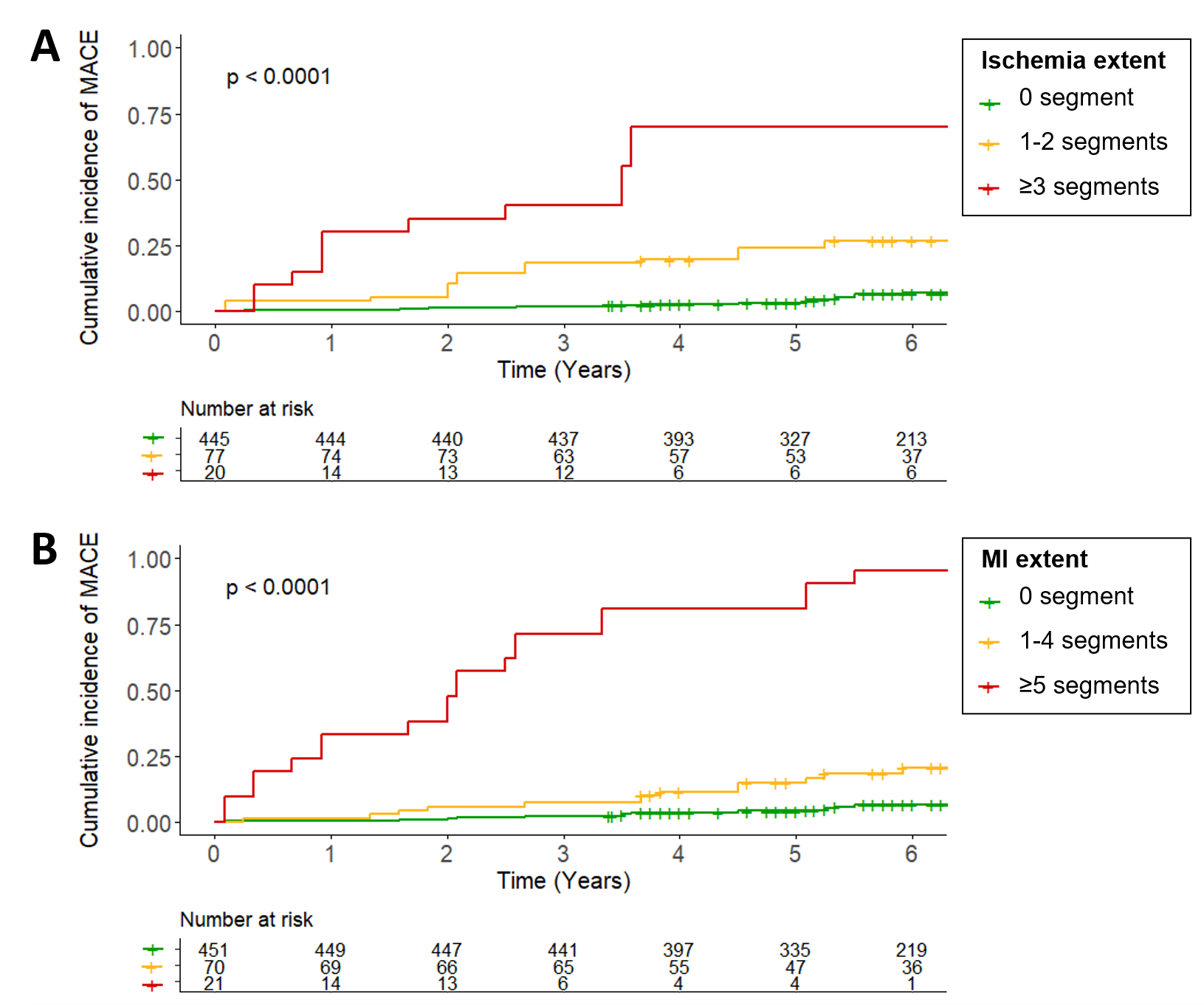
**

**SUPPLEMENTAL FILE 5**

**Univariable and multivariable analyses of silent myocardial ischemia for prediction of adverse events (N=542).**

|  | **Univariable analysis** | |  | **Multivariable analysis*** | |
| --- | --- | --- | --- | --- | --- |
|  | **Hazard Ratio**  **(95% CI)** | **p value** |  | **Hazard Ratio**  **(95% CI)** | **p value** |
|  |  |  |  |  |  |
| ***Primary outcome (MACE)*** |  |  |  |  |  |
| Cardiovascular mortality | 8.08 (4.21–15.5) | **<0.001** |  | 12.1 (5.11–28.7) | **<0.001** |
| Nonfatal MI | 8.99 (4.11–19.7) | **<0.001** |  | 14.5 (5.53–37.8) | **<0.001** |
|  |  |  |  |  |  |
| ***Secondary outcomes*** |  |  |  |  |  |
| All-cause of mortality | 2.33 (1.45–3.72) | **<0.001** |  | 1.66 (0.97–1.20) | 0.063 |
| Late coronary revascularization | 3.31 (1.30–8.41) | **0.012** |  | 4.68 (1.66–13.2) | **0.003** |
| Hospitalization for heart failure | 0.88 (0.42–1.88) | 0.749 |  | 0.67 (0.31–1.49) | 0.331 |
|  |  |  |  |  |  |

* Covariates in the model by stepwise variable selection with entry and exit criteria set at the p≤0.1 level for MACE: age, male, body mass index, hypertension, diabetes mellitus, dyslipidemia, current or previous smoking, LVEF per 10%, presence of unrecognized MI.

*Abbreviations*: CI: confidence interval; HR: hazard ratio; LVEF: left ventricular ejection fraction; MACE: major adverse cardiac events; MI: myocardial infarction.

**SUPPLEMENTAL FILE 6**

**Subgroup analysis.** Forest-plot of incidence of MACE based on the presence of silent ischemia in prespecified subgroups. *N events/N subgroup: number of patients who had a major adverse clinical event (MACE)/number of patients in the subgroup.


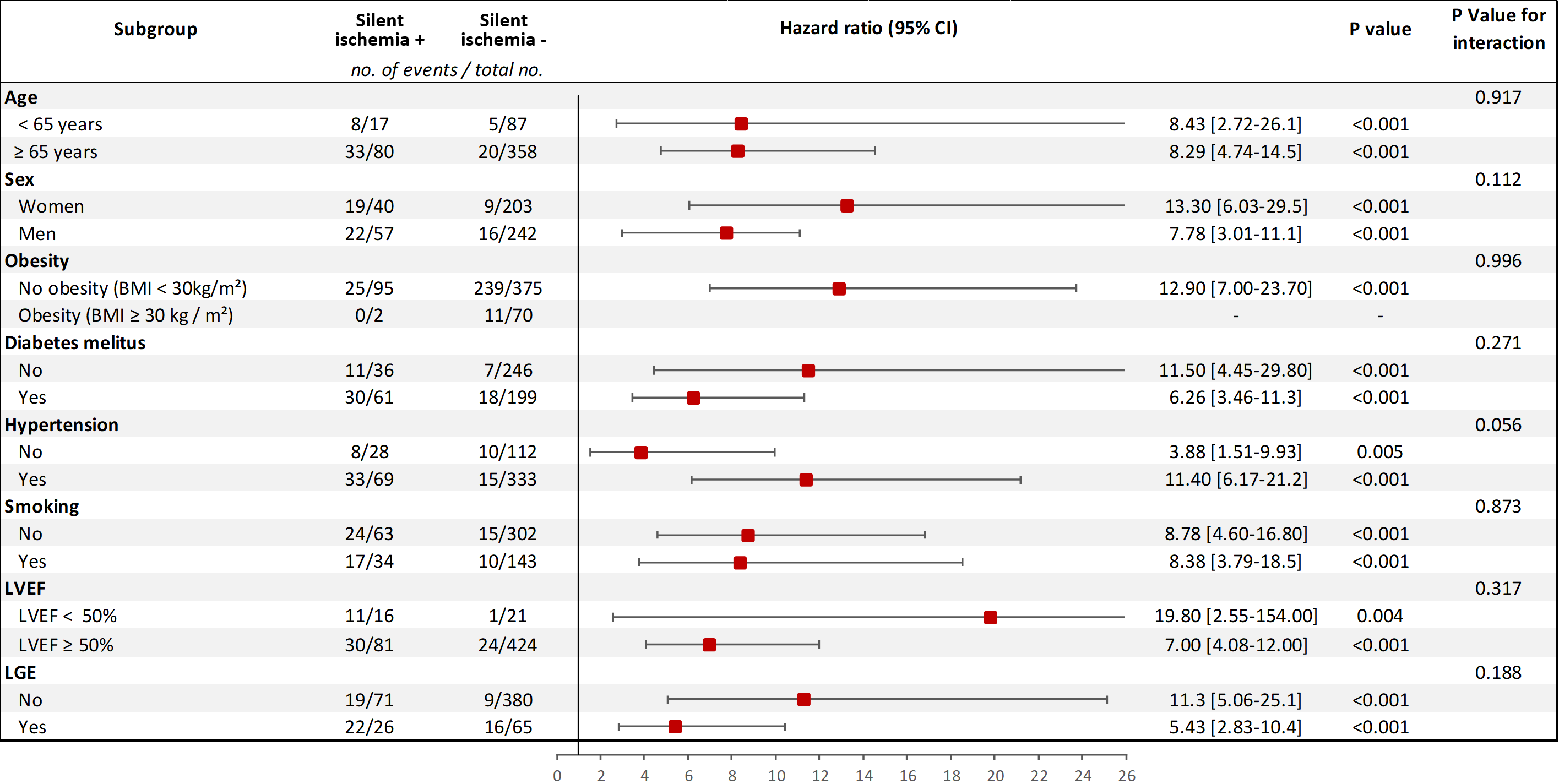


**SUPPLEMENTAL FILE 7**

**Multivariable Cox regression analysis for the prediction of MACE in patients with and without prior ischemic cryptogenic stroke using the propensity-matched population.**

|  | **Patients WITH**  **prior ischemic cryptogenic stroke**  **(N=542)** | |  | **Patients WITHOUT**  **prior ischemic cryptogenic stroke**  **(N=542)** | |
| --- | --- | --- | --- | --- | --- |
|  | **Hazard Ratio**  **(95% CI)** | **p value** |  | **Hazard Ratio**  **(95% CI)** | **p value** |
| Model 1* |  |  |  |  |  |
| Age | 1.00 (0.97-1.03) | 0.81 |  | 1.04 (1.01-1.06) | **0.03** |
| Male | 0.94 (0.57-1.54) | 0.80 |  | 1.19 (0.92-1.87) | 0.06 |
| Body mass index | 1.05 (0.97-1.13) | 0.23 |  | 1.07 (0.95-1.22) | 0.08 |
| Diabetes mellitus | 2.79 (1.51-5.15) | **<0.001** |  | 2.52 (1.30-4.97) | **<0.001** |
| Hypertension | 1.51 (1.23-2.04) | **<0.001** |  | 2.21 (1.18-4.21) | **<0.001** |
| Dyslipidemia | 0.61 (0.35-1.07) | 0.08 |  | 1.00 (0.77-1.31) | 0.98 |
| Current or previous smoking | 1.29 (0.76-2.17) | 0.35 |  | 0.97 (0.66-1.39) | 0.83 |
| LVEF, per 10% | 0.74 (0.59-0.93) | **0.011** |  | 0.89 (0.77-0.97) | **0.03** |
|  |  |  |  |  |  |
| Model 2^†^ |  |  |  |  |  |
| Presence of unrecognized MI | 8.44 (4.94-14.4) | **<0.001** |  | 1.98 (1.32-2.67) | **<0.001** |
|  |  |  |  |  |  |
| Model 3^‡^ |  |  |  |  |  |
| Presence of unrecognized MI | 9.53 (5.49-16.5) | **<0.001** |  | 1.77 (1.29-2.57) | **0.004** |
| Presence of silent ischemia | 12.4 (6.62-23.2) | **<0.001** |  | 3.72 (2.75-5.22) | **<0.001** |
|  |  |  |  |  |  |

* Covariates in the **model 1** by stepwise variable selection with entry and exit criteria set at the p≤0.2 level: age, male, hypertension, family history of CAD, LVEF per 10%, LV end-systolic volume index, per 10 ml/m^2^.

† Covariates in the **model 2**: model 1 with unrecognized MI.

‡ Covariates in the **model 3**: model 2 with silent ischemia.

*Abbreviations: BMI: body mass index; CAD: coronary artery disease; CI: confidence interval CV: cardiovascular; LGE: late gadolinium enhancement; MACE: major adverse cardiac events; LV: left ventricle; LVEF: left ventricular ejection fraction; MI: myocardial infarction.*
